# Communicability of varicella before rash onset: a literature review

**DOI:** 10.1017/S0950268821001102

**Published:** 2021-05-07

**Authors:** Mona Marin, Jessica Leung, Adriana S. Lopez, Leah Shepersky, D. Scott Schmid, Anne A. Gershon

**Affiliations:** 1 National Center for Immunization and Respiratory Diseases, Centers for Disease Control and Prevention, Atlanta, GA, USA; 2 Emory University, Atlanta, GA, USA; 3 Department of Pediatrics, Columbia University College of Physicians and Surgeons, New York, NY, USA

**Keywords:** Before rash onset, transmission, varicella, varicella-zoster virus, VZV

## Abstract

Varicella poses an occupational risk and a nosocomial risk for susceptible healthcare personnel and patients, respectively. Patients with varicella are thought to be infectious from 1 to 2 days before rash onset until all lesions are crusted, typically 4–7 days after onset of rash. We searched Medline, Embase, Cochrane Library and CINAHL databases to assess evidence of varicella-zoster virus (VZV) transmission before varicella rash onset. Few articles (7) contributed epidemiologic evidence; no formal studies were found. Published articles reported infectiousness at variable intervals before rash onset, between <1 day to 4 days prior to rash, with 1–2 patients for each interval. Laboratory assessment of transmission before rash was also limited (10 articles). No culture-positive results were reported. VZV DNA was identified by PCR before rash onset in only one study however, PCR does not indicate infectivity of the virus. Based on available medical literature, VZV transmission before rash onset seems unlikely, although the possibility of pre-rash, respiratory transmission cannot be entirely ruled out.

Primary infection with varicella-zoster virus (VZV) causes varicella (chickenpox), a highly contagious disease that can result in outbreaks in susceptible populations. Following primary infection, VZV remains latent in the body and may reactivate years or decades later to cause herpes zoster (HZ). VZV transmission poses an occupational risk and a nosocomial risk for healthcare personnel and susceptible patients, respectively [1–6]. Current guidance indicates that VZV is transmitted person-to-person, primarily by inhalation of aerosols from or direct contact with vesicular fluid of acute varicella or HZ skin lesions. Transmission could also potentially occur if infected respiratory tract secretions were aerosolised [7]. The incubation period for varicella is 10–21 days. Prodromal symptoms may be present, particularly in older children and adults; fever, malaise, anorexia, headache and occasionally mild abdominal pain may occur 1–2 days before the rash appears. Patients with varicella are generally thought to be infectious for 1–2 days before rash onset until all lesions are crusted, typically 4–7 days after onset of rash [7].

Airborne transmission of VZV has been suggested by some epidemiologic evidence indicating varicella in susceptible persons who had no direct contact with a varicella or HZ index patient and no other potential exposures to the virus [1, 4, 8, 9]. The mechanism by which VZV is spread from respiratory secretions and the relative importance of the respiratory route compared to spread from skin lesions, however, have been difficult to assess. VZV has long been a challenging virus to study in patients due to difficulty in propagating clinical samples in cell culture. Isolation of VZV from skin lesions was first accomplished by Weller and colleagues in 1956 [10]. Although difficult, it is possible to isolate VZV from patients with varicella, especially if skin vesicles are present. Interestingly, however, isolation of VZV from the respiratory tract is rare, even in patients with obvious varicella [11].

Implementation of effective infection control measures for varicella depends on understanding routes and timing of VZV transmission. We reviewed the medical literature to assess evidence for transmission of VZV before rash onset.

## Methods

We searched Medline, Embase, Cochrane Library and CINAHL databases for articles published in any language, from database inception through 31 October 2019, using the search terms (‘chickenpox’ or ‘varicella’ or ‘herpes zoster’) and (‘transmission’ or ‘spread’ or ‘epidemic’ or ‘isolation’) and (‘respiratory’ or ‘airborne’ or ‘nasal/pharyngeal/throat swab’ or ‘before rash’). The complete search strategy is described in Supplementary Table. Two authors independently reviewed each title and abstract to determine if the article likely included information on transmission of VZV before rash onset. For articles that passed this initial screen, we reviewed the full text; the summary of the full text review was reviewed by a second reviewer. We also reviewed references that described transmission of VZV identified in the reference sections of papers retrieved by the database search. Only articles that included original reports of assessment of VZV transmission before rash onset were retained.

We describe the evidence by whether it was provided using epidemiologic or laboratory data.

## Results

**Fig. 1.**
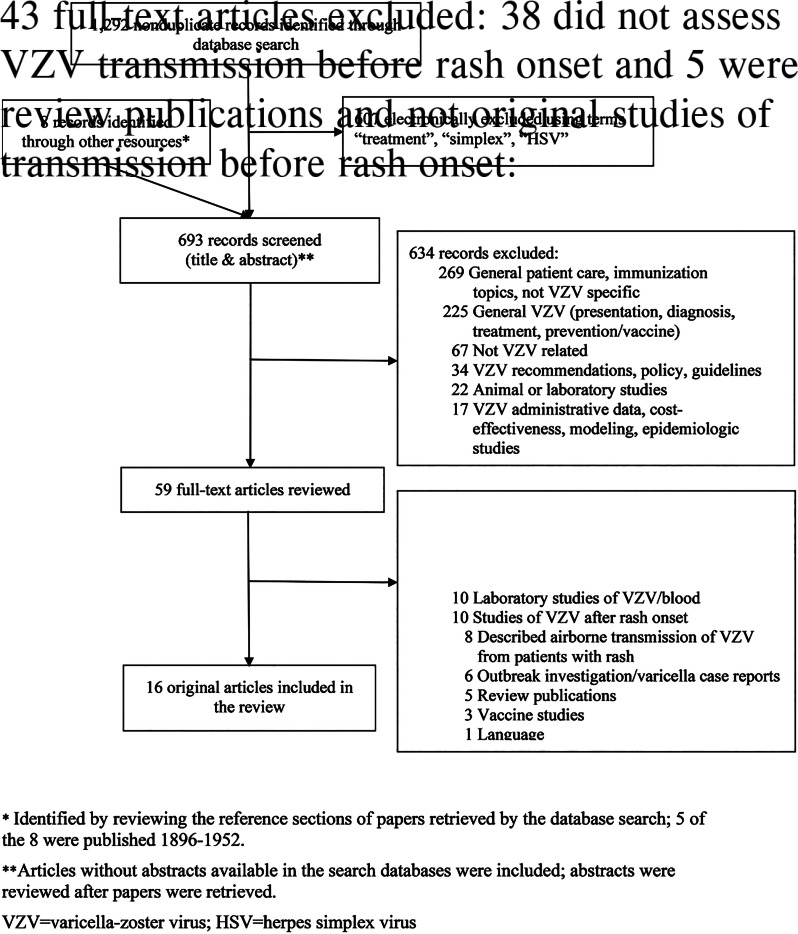
Study selection.

We screened 693 articles and identified 59 for full-text review; of these, 16 met inclusion criteria for our review (Fig. 1). Included articles originated from the United States (7), Japan (5), United Kingdom (2), Australia (1) and Czechoslovakia (1). Seven articles addressed VZV transmission before varicella rash onset using epidemiologic data [12–18] and 10 with laboratory data [11, 12, 19–26]; one epidemiologic study had a laboratory component [12].

### Epidemiologic evidence

Of the seven articles that addressed VZV transmission before rash onset with epidemiologic evidence, four were published during 1896–1940 and three during 1989–2003. Three contained data from reports of varicella case series during outbreaks in hospitals, three included institutional outbreak investigations (two in schools and one in a jail) and one was a case report (Table 1). Each report indicated transmission from an index patient who exposed contacts before being diagnosed with varicella (i.e. before rash onset); no reports described transmission from a patient with HZ before rash onset.

**Table 1.** Epidemiologic evidence of varicella-zoster virus (VZV) transmission before rash onset in patients with varicella, by timing of transmission in relation to rash onset

Study	Timing of VZV transmission in relation to rash onset	Description
Gordon and Meader [17]	<1 day before rash onset	Patient, age 4, removed (for a surgery then isolated in another unit) 9 h before varicella lesions were noted in another patient in the initial ward; removed patient developed varicella 17 days later.
Moore and Hopkins [14]	At least 1 day before rash onset	Outbreak investigation: excluding children with rash onset did not control the outbreak. Performed person-time analysis of in-class transmission from prodromal children in which exposure was defined as the day before a classmate stayed home with varicella. Cases of varicella were 3 times more likely to occur 12–17 days after exposure to a classmate considered during prodrome compared with children who did not have that exposure. Could not specifically identify the exact place of most exposures; transmission could have been from a playmate who was also a classmate or a sibling. Authors concluded that ‘the measure of association was too large to consider in-class exposure during the prodrome as unimportant.’
Schamberg and Kolmer [18]	At least 1 day before rash onset	Girl, age 16, developed a slight sore throat and a low fever, and was isolated in a room in a different floor of her home. A small whitish patch was noticed on the posterior pharyngeal wall. The next day the varicella rash appeared. Her brother, age 8, who was with the patient on the previous day, was kept in a distant part of the house. Sixteen days after exposure, the boy developed varicella. No information was given as to whether the brother left the home after exposure to his sister and potentially could have been exposed elsewhere.
Goodall and Washbourn [16]	At least 2 days before rash onset	Girl, age 4, had ‘chickenpox eruption’; her brother had been removed to hospital for scarlet fever 2 days earlier and admitted in a ward in which there had never previously been a case of varicella. The brother developed varicella 19 days after his sister.
Brunell [12]	At least 2 days before rash onset	Three siblings exposed to a parent with varicella were examined clinically and via nasopharyngeal and oropharyngeal specimens; all developed varicella. One child attended school; his rash was noted at noon during a school holiday; rash was not present the previous evening or in the morning when specimens were collected, therefore the child did not have lesions when he attended school the day before. Postcard inquiry of classmates on other potential exposures was negative. Varicella occurred in a school contact 17 days after exposure. VZV was not detected in specimens from nasopharyngeal and oropharyngeal specimens obtained daily from the 3 siblings from ~4 days before rash to 2 days after rash onset. Subsequent instances of transmission occurred in school. In 3 of the 4 cases that led to transmission, parents reported that the rash was discovered while the children were at home and denied that their children had attended school when the rash was present.
Evans [15]	At least 4 days before rash onset	Observation occurred in the context of an outbreak in a hospital with 27 cases among patients and staff during 3.5 months. A nurse who worked in a varicella ward was assigned for 2 h to work in a different, varicella-free, ward. She developed varicella 4 days later. She was not in the hospital on the day of rash onset. A patient confined to bed in the varicella-free ward developed varicella 16 days after the nurse's rash onset. The authors also report varicella in two nurses from the same ward 17 days and 18 days later; they had contact with the nurse who developed varicella during mealtimes from 1 day to 3 days before her rash onset.
Levy *et al*. [13]	‘A few days’ before rash onset	Three co-primary cases in inmates (rash onset within 8 days). Patients reported that each heard that another inmate's child who visited on the day of presumed exposure developed varicella a few days after the visit. During the visit session, the 3 patients were seated next to each other at adjacent tables. Despite further inquiry of inmates, visitors and visit centre staff, the putative source was not identified.

The authors reported that patients with varicella were infectious at various intervals before the rash appeared: <1 day (9 h) before (one report) [17], at least 1 day before (two reports) [14, 18], at least 2 days before (two reports) [12, 16], at least 4 days before (one report) [15]; one report described potential infectiousness ‘a few days’ before rash onset, but no source of exposure could be identified [13] (Table 1). Exposure of contacts before rash onset in the index patient was determined based on reports of index patients and contacts being in the same setting a number of days or hours before rash onset in the index patient and not while the index patient had rash: two reports described transmission among siblings, two in hospitals (ward contacts), two in schools (in one report parents denied that the child with varicella attended school when the rash was present and in a second, a person-time analysis, exposure was defined as the day before a classmate stayed home with varicella) and one in jail (putative source not identified). In one report, a patient with varicella who was considered to have transmitted the virus to a schoolmate was monitored daily clinically and with laboratory studies [12]. VZV was not detected in specimens of pharyngeal or nasal secretions obtained daily from ~4 days before to 2 days after rash onset (the same experience for his two younger siblings). However, VZV was detected (based on the characteristic cytopathic effect in cell culture) in vesicular fluid collected on days 2 and 3 of the rash in two of the three siblings [12].

### Laboratory evidence

Of the 10 articles that addressed VZV transmission before rash onset with laboratory evidence, five were published during 1966–1989; they reported results of virologic techniques (culture) to identify virus in the oropharynx [11, 12, 24–26]. Five articles were published during 1991–1999 and examined evidence of VZV DNA presence in the oropharynx using polymerase chain reaction (PCR) [19–23]. Four studies included exposed siblings, three included exposed patients, one study included exposed daycare contacts and one included children and young adult participants in the clinical trial for acyclovir (specimens collected <24 h after appearance of skin lesions (most were maculopapular)) (Table 2).

**Table 2.** Laboratory evidence for VZV presence in the oropharynx before and after rash onset in patients with varicella

Study	Method	Results of oropharyngeal specimen testing		Other results
		Before rash onset	After rash onset	
Gold [11]	Culture; specimens frozen and stored at −70°C before being tested. Study included exposed patients.	3/3 patients VZV negative; specimens collected daily from 9, 6 and 6 days before rash onset	5/5 patients VZV negative; specimens collected from day of rash onset to 3−5 days after	5/5 patients VZV positive; vesicular fluid collected within 3 days of rash onset; 23/25 specimens positive
Nelson and Geme [26]	Culture; most specimens stored at −50 °C for 1–2 weeks before inoculation. Source of patients not indicated.	45 specimens from 29 children tested VZV negative; specimens collected from 8 days before to 4 days after rash onset (one specimen was collected 3 days before and four were collected 1 day before)		1/1 patient VZV positive; vesicular fluid from herpes zoster lesions, repetitive isolation
Trlifajova *et al*. [25]	Culture; specimens inoculated at most 1 h incubation at room temperature in collecting medium. Study included exposed patients.	57/57 nasal and 56/56 oropharyngeal specimens collected during the entire incubation period VZV negative (11 patients); 11 nasal and 11 oropharyngeal specimens were collected in the last 3 days before rash onset	3/23 pharyngeal and 2/22 nasal specimens VZV positive during the first 3 days after rash onset; none of the 13 samples collected on the first day of rash tested positive	11/11 patients VZV positive; vesicular fluid collected during the first 3 days after rash onset (12/12 specimens)
Ozaki *et al*. [24]	Culture; specimens inoculated at most 4 h incubation at 4 °C in collecting medium. Study included paediatric outpatient patients with varicella, of whom 5 children during the incubation period, exposure source unknown.	5/5 patients VZV negative; specimens collected 6, 4, 2 and 1 day(s) before rash onset	5/117 patients with varicella VZV positive; specimens collected 1–2 days after rash onset	>90% VZV positivity rate; vesicular fluid collected during the first 3 days after rash onset
Brunell [12]	Culture; swabs placed in partially frozen (4 °C) medium; up to 30 min between collection of specimens and inoculation. Study included children exposed to a parent with varicella.	3/3 patients VZV negative; nasopharyngeal and oropharyngeal specimens obtained daily from ~4 days before rash onset	3/3 patients VZV negative; nasopharyngeal and oropharyngeal specimens obtained daily on days 1 and 2 after rash onset	2/3 patients VZV positive; vesicular fluid collected on days 2 and 3 of the rash
Koropchak *et al*. [22]	PCR; detection limit = 45 copies. Culture. Study included immunocompetent children and adults participants in a clinical trial evaluating oral acyclovir for treatment of varicella.		PCR: 1/30 patients VZV positive; specimens collected <24 h (mean 15.9 h) after appearance of skin lesions (most maculopapular); the VZV-positive patient had oral lesions typical of VZV Culture: 0/19 patients VZV positive during same collection interval	Skin lesion specimens collected <24 h after rash onset: PCR: 21/28 patients VZV positive Culture: 5/24 patients VZV positive
Ozaki *et al*. [23]	PCR; detection limit = 50 copies. Study included exposed siblings.	11/42 specimens collected from 15 days before rash onset VZV positive (18 children); 4/9 specimens collected within 5 days before rash onset VZV positive (3/7 children). A less sensitive assay (5 × 10^3^ copies) did not detect viral DNA	90% specimens VZV positive during the first 3 days after rash onset (39 specimens); 26% VZV positive with the less sensitive assay	
Hondo *et al*. [21]	PCR; estimated number of copies by comparing with the standard curves. Study included exposed siblings.	Two patients; throat swabs; DNA in low titres during the entire incubation period (<2 copies/swab); moreover, within 3 days before rash onset, VZV DNA detected only in 1/6 specimens	Titres abruptly increased to high levels when rash occurred; levels >100-fold higher than during the incubation period	
Asano *et al*. [19]	PCR; detection limit = 100 copies. Study included exposed siblings.	0/2 patients VZV DNA positive in throat swabs collected daily from 8 days before rash onset or on the day of rash onset	2/2 patients VZV DNA positive in throat swabs for 4 days starting the day after rash onset	
Asano *et al*. [20]	PCR; detection limit = 100 copies. Study included daycare contacts.		1/13 children VZV DNA positive; throat swabs collected 4 h after recognised onset of varicella in a contact (only a few maculopapular lesions)	All children were vaccinated postexposure and none developed varicella (including the one with the VZV DNA positive throat swab). Several environmental samples collected at the same time as the throat swabs were positive

None of the studies that used culture to identify VZV in oropharyngeal specimens reported detection of the virus during the varicella incubation period. While these studies included a limited number of children who were tested before developing the characteristic varicella rash (between 23 and 27), the number of oropharyngeal specimens collected during the entire incubation period was ~160; ~47 were collected during the 3 days before rash onset (Table 2). These include the child mentioned above [12] considered the source for a schoolmate who was VZV negative in culture of specimens of pharyngeal or nasal secretions collected before rash onset. Low levels of positivity (8–11%) [24, 25] were reported in oropharyngeal samples collected after rash onset, with some children having lesions in the mouth [24]. Conversely, using the same methods, the studies reported positivity >90–100% in skin lesion specimens collected within the first 3 days after rash onset. Additionally, one study reported no positive cultures among 19 patients when oral specimens were collected <24 h (mean 15.9 h) after the appearance of skin lesions (most maculopapular) [22].

Positivity of oral specimens collected before rash onset was reported when VZV PCR was used for testing, although in rare instances (Table 2). On a limited number of patients or samples tested, Ozaki reported that three of seven patients and four (44%) of nine specimens collected within 5 days before rash onset were VZV DNA positive; two VZV-positive specimens were collected 5 days before rash onset and two were collected 2 days before [23]. One specimen collected 1 day before rash onset and another one collected 2 days before rash onset were negative. A less sensitive assay (5 × 10^3^ copies) did not detect viral DNA in this study. Conversely, 90% of throat specimens (*n* = 39) collected during the first 3 days after rash onset were PCR positive (100% of patients, *n* = 18). Asano reported VZV DNA in throat specimens of one child of 13 tested (susceptible), with the specimens collected 4 h after recognising onset of varicella in a contact who had only a few maculopapular lesions [20]. This result was considered indirect evidence of excretion of VZV from the index case, assumed to be from saliva. Lastly, in oropharyngeal specimens collected <24 h after appearance of the rash (most skin lesions were maculopapular), Koropchak reported VZV DNA present in one patient of 30 tested [22]. The VZV-positive patient had oral lesions typical of VZV mucosal infection. This compares with 75% PCR positivity rate in skin lesion specimens collected during the same period. Two other studies reported negative results in four patients with daily collection of oropharyngeal specimens within 5 days before rash onset [19, 21].

## Discussion

This review confirms the scarcity of evidence in the medical literature on transmission of VZV before varicella rash onset and substantiates limitations on reaching definitive conclusions about VZV transmission before appearance of skin lesions.

Few reports contributed epidemiologic evidence; no formal studies have been published, and most information was anecdotal and reported decades ago. The epidemiologic investigations reported that patients with varicella were infectious at various intervals before rash onset, ranging from <1 day to 4 days before, with only 1–2 patients identified for each interval. One study that included more cases concluded that transmission during the incubation period occurred based on person-time analysis of in-class transmission, where exposure was defined as the day before a classmate stayed home with varicella [14]. The epidemiologic reports are based on information from healthcare personnel and parental observations about when/if rash was present. Some rashes may not have been identified soon enough to rule out transmission from skin lesions. Additionally, observations occurred in the context of the wide circulation of the virus; exposures could have occurred from other sources. Studies that observed no evidence of pre-onset transmission have been reported, but we did not systematically review them for this report. Several outbreak investigations in healthcare facilities reported potential exposure prior to rash onset in a patient with varicella but no transmission among exposed staff and patients [5, 27]. A report that indicated infectiousness <1 day before rash onset also indicated that transmission did not occur if exposure was ⩾1 day before rash onset (18 susceptible children exposed 1–2 days before rash onset in contacts) [17].

Laboratory evidence supporting transmission of VZV before rash onset is also very limited. No culture-positive results were reported. PCR was a major advance for investigating disease caused by VZV, proving much easier, cheaper and more sensitive than culture of VZV. VZV DNA was found in the skin, saliva and respiratory tract of patients with varicella and HZ when rash was present [28, 29]. VZV DNA was identified by PCR during the incubation period in only one study [23]. While the proportion of detection appears high (44%) in that study, it was based on four of nine specimens collected from seven patients within 5 days before rash onset (VZV DNA identified in three patients). Two other studies reported a low PCR positivity rate when specimens were collected within 24 h of rash onset: two of 43 patients tested positive, one of whom had oral varicella lesions [20, 22]. Methods for specimen collection and storage varied among studies and may have been suboptimal (collection using cotton swabs, freezing of specimens). It is critical to recognise, however, that presence of VZV DNA does not necessarily indicate infectivity or transmissible virus. For example, VZV DNA was found to persist in the environment of varicella and HZ patients’ rooms up to a month post rash onset [9, 30].

Varicella is a highly contagious viral disease. Many viral infections are spread by the respiratory route and virus can be readily identified in respiratory secretions of infected patients. Nevertheless, in patients with varicella, isolation of VZV in the respiratory tract has rarely been reported. Coughing and sneezing, associated with respiratory transmission of infectious diseases, are not characteristic of varicella. On the other hand, the pruritic vesicles characteristic of varicella contain high levels of well-formed, highly infectious virions [31] and itching of skin lesions is common during varicella. The virus was easily detected (75–100% for both culture and PCR) in skin lesion specimens collected within 3 days after rash onset. One study reported DNA values >100-fold higher in oropharyngeal samples when rash occurred compared to during the incubation period (titres during the incubation period were estimated at <2 copies/swab) [21]. These observations may indicate that failure to demonstrate VZV in respiratory secretions in most patients could result from lack of replication of the virus in the oropharynx at levels that enable identification and spread. However, low copy number during the incubation period does not preclude transmissibility.

Based on the available medical literature, VZV transmission seems unlikely prior to rash onset; however, it is impossible to prove it never happens. We did not find conclusive evidence to alter existing infection control guidelines. Providers should continue to implement appropriate infection control measures since the possibility of pre-rash, respiratory transmission of VZV cannot be entirely ruled out and sufficiently early identification of the rash, when only a few lesions are present, might be missed. Considering the scarcity and age of the existing evidence, additional evidence, using current laboratory assays would be beneficial. These studies can be undertaken in regions where varicella infections are still high; varicella rates are now low in the United States due to widespread use of the varicella vaccine [32] and situations of exposure occur less commonly today.

## Data Availability

All data presented in this review are from previously published papers and available from the cited references.

## Supplementary material

For supplementary material accompanying this paper visit http://dx.doi.org/10.1017/S0950268821001102. click here to view supplementary material
