# Supplementary material for: Communicability of varicella before rash onset: a literature review
Source: Epidemiol Infect. 2021 May 7;149:e131. doi: 10.1017/S0950268821001102 (PMC8193770; doi:10.1017/S0950268821001102)
Supplement: Supplementary file 1 [file S0950268821001102sup001.docx]

**Epidemiology and Infection**

**Communicability of varicella before rash onset: a literature review**

**Authors:** Mona Marin, MD, Jessica Leung, MPH, Adriana S. Lopez, MSH, Leah Shepersky, D. Scott Schmid, PhD, Anne A. Gershon, MD

**Supplementary Material**

**Supplementary Table: Search Strategy**

Below are the terms used in the systematic literature search. Search included articles without abstracts available in the databases.

Chickenpox OR chicken-pox OR varicella* OR “herpes zoster” OR shingles OR “herpesvirus 3” OR VZV

AND

Transmission OR transmit* OR spread OR infect* OR outbreak* OR epidemi* OR isolat*

AND

Respirat* OR airborne OR air-borne OR aerosol* OR nasal OR pharyn* OR nasopharyn* OR throat OR swab OR (before ADJ5 rash*) OR (prior ADJ5 rash*) OR (before ADJ5 exanthem*) OR (prior ADJ5 exanthem*)

NOT

exp animals/ not exp humans/

Our search yielded the following results by database:

| **Database** | **Year Database Coverage Began** | **Citations Retrieved*** | **Date Run** |
| --- | --- | --- | --- |
| Medline (OVID) | 1946 | 1,062 | 11/04/2019 |
| Embase (OVID) | 1947 | 152 | 11/04/2019 |
| Cochrane Libraries | 1800 | 88 | 11/04/2019 |
| CINAHL (Ebsco) | 1981 | 54 | 11/04/2019 |
| Total |  | 1,356 |  |

*Totals before duplicates were removed.
